# Mode of recording and modulation frequency effects of auditory steady state response thresholds^☆^

**DOI:** 10.1016/j.bjorl.2015.12.005

**Published:** 2016-03-29

**Authors:** Bahram Jalaei, Moslem Shaabani, Mohd Normani Zakaria

**Affiliations:** a Universiti Sains Malaysia, School of Health Sciences, Audiology Programme, Kelantan, Malaysia; b Iran University of Medical Sciences, Department of Audiology, Tehran, Iran; c University of Social Welfare and Rehabilitation Sciences, Department of Audiology, Tehran, Iran

**Keywords:** Auditory steady state response, Hearing threshold, Contralateral recording, Ipsilateral recording, Resposta auditiva de estado estável, Limiar auditivo, Registro contralateral, Registro ipsilateral

## Abstract

**Introduction:**

The performance of auditory steady state response (ASSR) in threshold testing when recorded ipsilaterally and contralaterally, as well as at low and high modulation frequencies (MFs), has not been systematically studied.

**Objective:**

To verify the influences of mode of recording (ipsilateral *vs*. contralateral) and modulation frequency (40 Hz *vs*. 90 Hz) on ASSR thresholds.

**Methods:**

Fifteen female and 14 male subjects (aged 18–30 years) with normal hearing bilaterally were studied. Narrow-band CE-chirp^®^ stimuli (centerd at 500, 1000, 2000, and 4000 Hz) modulated at 40 and 90 Hz MFs were presented to the participants’ right ear. The ASSR thresholds were then recorded at each test frequency in both ipsilateral and contralateral channels.

**Results:**

Due to pronounced interaction effects between mode of recording and MF (*p* < 0.05 by two-way repeated measures ANOVA), mean ASSR thresholds were then compared among four conditions (ipsi-40 Hz, ipsi-90 Hz, contra-40 Hz, and contra-90 Hz) using one-way repeated measures ANOVA. At the 500 and 1000 Hz test frequencies, contra-40 Hz condition produced the lowest mean ASSR thresholds. In contrast, at high frequencies (2000 and 4000 Hz), ipsi-90 Hz condition revealed the lowest mean ASSR thresholds. At most test frequencies, contra-90 Hz produced the highest mean ASSR thresholds.

**Conclusions:**

Based on the findings, the present study recommends two different protocols for an optimum threshold testing with ASSR, at least when testing young adults. This includes the use of contra-40 Hz recording mode due to its promising performance in hearing threshold estimation.

## Resumo

**Introdução:**

O desempenho da resposta auditiva de estado estável (RAEE) em testes de limiar com registros ipsilateral e contralateral, e modulações em frequências (MFs) não tem sido sistematicamente estudados.

**Objetivo:**

Verificar a influência do modo de registro (ipsilateral *vs.* contralateral) e da modulação em frequências (40 Hz *vs.* 90 Hz) nos limiares de RAEE.

**Método:**

Foram estudados 15 mulheres e 14 homens (18-30 anos) com audição bilateral normal. Estímulos CE-chirp^®^ de banda estreita (centrados em 500, 1.000, 2.000 e 4.000 Hz) modulados em 40 e 90 Hz de MF foram apresentados à orelha direita dos participantes. Em seguida, os limiares de RAEE foram registrados em cada frequência de teste nos canais ipsilateral e contralateral.

**Resultados:**

Devido aos pronunciados efeitos de interação entre o modo de registro e MF (p *< *0,05 por variância com dois fatores para medidas repetidas–ANOVA duas vias), os limiares médios de RAEE foram então comparados entre quatro condições (Ipsi-40 Hz, Ipsi-90 Hz, Contra-40 Hz e Contra-90 Hz), com o uso de variância e com um fator para medidas repetidas (ANOVA uma via). Nas frequências de teste de 500 e 1.000 Hz, a condição Contra-40 Hz produziu os mais baixos limiares médios de RAEE. Em contraste, em altas frequências (2.000 e 4.000 Hz), a condição Ipsi-90 Hz revelou os mais baixos limiares médios de RAEE. Na maioria das frequências de teste, a condição Contra-90 Hz produziu os mais elevados limiares médios de RAEE.

**Conclusões:**

Com base nos achados do presente estudo, os autores recomendam dois protocolos diferentes para a realização de um teste de limiares ideal com RAEE, pelo menos em adultos jovens. Isso inclui o uso do modo de registro Contra-40 Hz, devido ao seu desempenho promissor nas estimativas do limiar auditivo.

## Introduction

Auditory steady state response (ASSR) is an electrical potential evoked by periodic amplitude modulated and/or frequency modulated stimuli. Differently from conventional auditory evoked potentials, ASSR utilizes an objective threshold detection method, which provides clinicians and researchers a convenient way for estimating behavior hearing thresholds.

The ASSR thresholds have been shown to be closely related to pure tone audiogram in various studies. In fact, ASSRs evoked by stimuli at 90 and 40 Hz modulation frequencies (MFs) show consistency with auditory brainstem response (ABR) and activity in the upper region of central auditory nervous system (CANS), respectively.^1^, ^2^, ^3^, ^4^ That is, similar to ABR, 90 Hz ASSR is the choice for estimating auditory sensitivity at mid and high frequencies. The 40 Hz ASSR, however, performs like the cortical auditory evoked potential (CAEP), which is suitable for determining auditory sensitivity at low frequencies.^5^, ^6^

The effects of mode of recording (*i.e.*, ipsilateral *vs*. contralateral) on ASSR have also been investigated. Van Maanen and Stapells^7^ conducted a study on younger (≤6 months) and older (>6 months) infants with normal hearing bilaterally. They recorded ASSR threshold at 500, 1000, 2000, and 4000 Hz carrier frequencies (CFs) with MFs of between 81 and 101 Hz. Both ipsilateral and contralateral recordings were obtained at each CF. They then found that the ipsilateral recording yielded better ASSR thresholds than contralateral recording at all CFs. Consequently, they recommended the use of only ipsilateral recording in ASSR threshold determination, at least when testing infants. On the other hand, Kaf and Danesh^8^ conducted a study comparing the ipsilateral and contralateral recordings of ASSR among healthy female adults. The ASSRs were recorded at an intensity level of 65 dB SPL at CFs of 500, 2000, and 4000 Hz and at MFs of 39 and 79 Hz. They then found no significant differences of ASSR amplitudes and latencies between the ipsilateral and contralateral conditions at both MFs.

Typically, the ASSR testing is carried out to estimate behavioral hearing thresholds. To the best of authors’ knowledge, the performance of ipsilateral and contralateral recordings of ASSR at threshold levels in adults has not been systematically studied, particularly at low and high MFs. With regard to the study by Kaf and Danesh,^8^ the ASSRs were only determined at one fixed supra-threshold level. The ASSR outcomes at threshold levels, nevertheless, are unclear and might show some differences with the supra-threshold findings. In line with this, Vander Werff and Brown^9^ found that the ASSRs recorded at supra-threshold levels (as shown by the amplitude growth functions) were not advantageous in estimating behavioral thresholds in the tested groups. Thus, the present study aimed to compare ASSR thresholds between contralateral and ipsilateral recordings at 40 and 90 Hz MFs in healthy young adults.

## Methods

### Participants

This study employed descriptive and repeated measures design. Fifteen female and 14 male participants with age ranging from 18 to 28 years (mean of 23.4 ± 2.65 years), right-handed, with normal hearing in both ears (thresholds ≤15 dB HL from 500 to 4000 Hz) and without any record of head injuries and neurological disorders or any active ear pathology took part in the study. All of them agreed to voluntary participation by signing the written informed consent. Prior to testing, an ethical approval from the respective institution was obtained, in accordance with the Declaration of Helsinki (USM/PPP/JEPeM [245.3(5)]).

### Equipment and stimuli

To ensure normal hearing status, basic audiologic evaluations including otoscopy, admittance audiometry (Model AZ26, by Interacoustic) and pure tone audiometry (Model AC 40 two-channel audiometer) were performed on all participants in a sound-treated room within the Audiology Clinic, University Hospital.

ASSR thresholds were then recorded with a two-channel Eclipse system (Interacoustic Corporation, Denmark) and narrow-band CE-chirp^®^ signals centered at 500, 1000, 2000, and 4000 Hz were used as the test stimuli. The chirp stimuli were designed to compensate for the cochlear delay and to produce a bigger response.^10^ The amplitude and frequency modulation depths were 100% and 20% (±10%), respectively. For each test stimulus, the modulation frequencies were 40 and 90 Hz. The stimulus level was calibrated in dB nHL. The common mode rejection (CMR) ratio of pre-amplifier was more than 115 dB at any frequency. The number of epochs and time analysis were 16 and 1 s, respectively. The recorded responses were amplified 100,000 times and filtered using a band-pass of 0.1–100 Hz (12 dB/octave).

Four scalp electrodes were placed on the participant's head: non-inverting on the forehead, inverting on each mastoid, and ground on the cheek. For both ipsilateral and contralateral recordings, the insert earphone was placed only in the right ear (*i.e.*, ASSRs recorded in the right and left channels represented ipsilateral and contralateral conditions, respectively). The impedance of electrodes was maintained to be less than 3 kΩ throughout the measurements.

### ASSR procedure

After giving the proper instructions to the participants, the ASSR testing began. While lying comfortably on a test bed, the stimuli were presented to the right ear of the participants using the multiple auditory steady state response (MASTER) technique through the insert earphone. This technique offers a time-effective way for stimulus presentation, as four frequencies can be tested simultaneously. The measurement started with 40 Hz MF and was followed by 90 Hz MF recordings, as the participants were typically awake at the beginning of the testing. Nevertheless, during the 40 Hz ASSR measurements, the participants’ state was closely monitored to ensure adequate wakefulness.

For detecting the response in a quick and accurate manner, the ASSR Eclipse system utilized the ‘Full Spectrum Detection Engine’ method. With this method, amplitude and phase coherence components were combined, and responses from higher harmonics were also included in the detection algorithm. In comparison to the use of information only from the first harmonic, significantly higher response detection rates and shorter detection times were observed when all available responses (amplitude and phase) from first and higher harmonics were utilized.^11^

With the ASSR Eclipse device, at a particular intensity level, the response is considered present if it reaches an amplitude level that is within 95% confidence within the default time of 6 min. In the current study, the ASSR measurement started at 60 dB nHL of intensity. If the response was clearly detected (reached 95% confidence) sooner than the default time, the trial was stopped and the stimulus intensity was decreased by 10 dB. If the confidence of response was less than 50% during the first 3 min, the trial was stopped and the test was repeated at a similar level. If unclear response was still observed (<50% confidence), the trial was stopped and the stimulus intensity was increased by 5 dB. The measurements continued until ASSR threshold was obtained. The ASSR threshold was defined as the lowest intensity level that elicited response with 95% confidence in 6 min. At the threshold level, the measurements were repeated twice to confirm the test reproducibility. The ASSR threshold was obtained at each test frequency at different MFs and for both recording conditions. To avoid fatigue during the recording, 10 min of break was given to the participants between each trial.

### Statistical analysis

For data analysis, both descriptive and inferential statistics were used. Mean and standard deviation (SD) were expressed as applicable. All the numerical data were found to be normally distributed as shown by Kolmogorov–Smirnov test (*p* > 0.05). For each test frequency, two-way repeated measures ANOVA (with mode of recording and MF as the factors) was performed to compare mean ASSR thresholds between ipsilateral and contralateral recordings, as well as between 40 Hz and 90 Hz MFs. To analyze the simple main effects (in the conditions where significant interaction effects between factors were observed in the two-way ANOVA analysis), one-way repeated measures ANOVA was conducted to compare mean ASSR thresholds among the following conditions: ipsi-40 Hz, contra-40 Hz, ipsi-90 Hz, and contra-90 Hz. Prior to this, the Mauchly test was carried out to test the assumption of sphericity. For pairwise comparisons, Bonferroni correction was used. The statistical significance level was set at *p* < 0.05. All data were analyzed using the SPSS software v. 20 (SPSS Inc., Chicago, IL, United States).

## Results

Mean and standard deviation of ASSR thresholds for both recording modes at different MFs at specific test frequencies are shown in Table 1. Descriptively, except at the 4000 Hz test frequency, contra-90 Hz condition revealed the highest mean ASSR threshold at all test frequencies. At the 4000 Hz test frequency, the highest mean ASSR threshold was noted in ipsi-40 Hz condition. At 500 and 1000 Hz test frequencies, the lowest mean ASSR thresholds were noted in contra-40 Hz condition. This is followed by ipsi-40 Hz condition, which showed slightly higher mean ASSR thresholds than that of contra-40 Hz condition (4.2 and 1.4 dB higher at 500 and 1000 Hz, respectively). Whereas at 2000 and 4000 Hz test frequencies, ipsi-90 Hz condition produced the lowest mean ASSR thresholds. The second lowest mean ASSR thresholds were found in contra-40 Hz condition (0.2 and 2.1 dB higher than ipsi-90 Hz condition at 2000 and 4000 Hz, respectively).

**Table 1:** Descriptive and inferential statistical analyses of ASSR thresholds (in dB nHL) for different modes of recordings and modulation frequencies at specified test frequencies.

Test frequency	ASSR threshold (mean ± SD) (dB nHL)				*p*-Value		
	Ipsilateral		Contralateral				
	40 Hz MF	90 Hz MF	40 Hz MF	90 Hz MF	Mode	MF	Mode × MF
500 Hz	22.6 ± 11.2	27.6 ± 12.4	18.4 ± 7.6	35.9 ± 12.3	0.359	<0.05^a^	<0.05^a^
1000 Hz	15.9 ± 6.4	18.3 ± 8.9	14.5 ± 5.7	26.6 ± 12.9	0.048^a^	<0.05^a^	<0.05^a^
2000 Hz	19.7 ± 7.7	16.2 ± 7.4	16.4 ± 6.3	21.2 ± 11.7	0.651	0.562	<0.05^a^
4000 Hz	20.3 ± 5.8	16.0 ± 7.8	18.1 ± 6.0	19.7 ± 11.6	0.708	0.216	0.010^a^

MF, modulation frequency.

a Significant at *p* < 0.05.

To confirm these descriptive observations, two-way repeated measures ANOVA was conducted; the statistical outcomes are shown in Table 1. As shown, significant interaction effects (between mode of recording and MF) were found at all test frequencies (*p* < 0.05). Consequently, the interpretation of the main effects (*i.e.*, the effects of mode of recording and MF on ASSR thresholds) can be misleading. To address this issue, one-way repeated measures ANOVA was carried out and the simple main effects were determined. At all test frequencies, Mauchly's test of sphericity revealed that the sphericity had not been violated (*p* > 0.05). Consequently, correction of the degrees of freedom was not required. Table 2 reveals the statistical outcomes of this analysis. As shown, the mean ASSR thresholds among the conditions (*i.e.*, ipsi-40 Hz, contra-40 Hz, ipsi-90 Hz, and contra-90 Hz) were found to be statistically different from each other at all test frequencies (*p* < 0.05).

**Table 2:** *p*-Values for one-way repeated measures ANOVA and pairwise comparisons at each test frequency.

Statistical test	Test frequency (Hz)			
	500	1000	2000	4000
*One-way ANOVA*	0.001^a^	0.001^a^	0.007^a^	0.036^a^

*Pairwise comparison*				
Ipsi-40 Hz *vs*. contra-40 Hz	0.171	0.966	0.027^a^	0.209
Ipsi-90 Hz *vs*. contra-90 Hz	0.014^a^	0.001^a^	0.014^a^	0.240
Ipsi-40 Hz *vs*. ipsi-90 Hz	0.674	1.000	0.114	0.007^a^
Contra-40 Hz *vs*. contra-90 Hz	0.001^a^	0.001^a^	0.106	1.000
Contra-40 Hz *vs*. ipsi-90 Hz	0.003^a^	0.382	1.000	0.892
Contra-90 Hz *vs*. ipsi-40 Hz	0.001^a^	0.001^a^	1.000	1.000

a Significant at *p* < 0.05.

The pairwise comparisons using Bonferroni correction at each test frequency was then performed; the results are shown in Table 2. At the 500 Hz test frequency, statistically significant results were obtained in ‘ipsi-90 Hz *vs*. contra-90 Hz’, ‘contra-40 Hz *vs*. contra-90 Hz’, ‘contra-40 Hz *vs*. ipsi-90 Hz’ and ‘contra-90 Hz *vs*. ipsi-40 Hz’ conditions (*p* < 0.05). At the 1000 Hz test frequency, three conditions (*i.e.*, ‘ipsi-90 Hz *vs*. contra-90 Hz’, ‘contra-40 Hz *vs*. contra-90 Hz’ and ‘contra-90 Hz *vs*. ipsi-40 Hz’) produced statistically significant outcomes (*p* < 0.05). At both the 500 and 1000 Hz test frequencies, the mean ASSR thresholds were statistically different between contra-40 Hz and contra-90 Hz conditions. This supports the earlier observation that the contra-40 Hz condition produced the lowest mean ASSR threshold, while the contra-90 Hz condition revealed the highest mean ASSR threshold.

At the 2000 Hz test frequency, only one condition (*i.e.*, ‘ipsi-40 Hz *vs*. contra-40 Hz’) revealed a significant result (*p* = 0.027). Similarly, at the 4000 Hz test frequency, only one condition (*i.e.*, ‘ipsi-40 Hz *vs*. ipsi-90 Hz’) produced a statistically significant outcome (*p* = 0.007).

## Discussion

### Comparison between low and high modulation frequencies

Recall that the present study aimed to compare the ASSR thresholds recorded at high and low modulation frequencies, as well as between ipsilateral and contralateral recordings in healthy adults. At the 500 and 1000 Hz test frequencies, no significant differences in ASSR threshold were found between ipsi-40 Hz and ipsi-90 Hz conditions. However, contra-90 Hz condition produced significantly higher ASSR thresholds than that of contra-40 Hz condition. The weak efficiency of ASSR at low frequencies and at high MF found in the present study is in line with previous studies.^12^, ^13^, ^14^ The possible reason is that the ASSR at high MF is generated predominantly by the auditory brainstem. Consequently, it shows almost similar performance with ABR in the threshold testing.^15^, ^16^ Elevated ABR thresholds at low frequencies have been well documented and are related to poorer neural synchrony.^17^, ^18^

At the 500 and 1000 Hz test frequencies, lower mean ASSR thresholds were observed at 40 Hz MF than at 90 Hz MF. In this situation, 40 Hz ASSR seems to be superior to 90 Hz ASSR, since it produced lower thresholds (perhaps closer to the behavioral hearing thresholds). The superiority of ASSR at low MF is consistent with the previous reports.^6^, ^14^ van der Reijden et al.^14^ conducted a study to compare thresholds between tone burst ABR (t-ABR) and ASSR (at 40 and 90 Hz MFs) at 500 and 2000 Hz CFs. They then found that 40 Hz ASSR yielded the lowest thresholds, particularly at 500 Hz CF. ASSR at low MF is generated by the auditory midbrain, thalamus, and primary auditory cortex.^19^ If compared with high MF ASSR (*i.e.*, predominantly generated by the auditory brainstem), lower ASSR thresholds at 40 Hz MF is possibly due to increased neural connections and binaural activities within these upper regions of CANS.^4^ Furthermore, since female subjects were also included in the present study, the superiority of ASSR at 40 Hz MF might also be influenced by a hormonal factor. Estrogen, the primary sex steroid for females, is known to affect GABAergic transmission that modulates ASSR amplitudes (see Zakaria et al.^20^ and Griskova-Bulanova et al.^21^ for detailed discussions).

At 2000 and 4000 Hz test frequencies, the mean ASSR thresholds found in the present study were descriptively lower in ipsi-90 Hz condition than in ipsi-40 Hz condition. This difference was then found to be statistically significant only at the 4000 Hz test frequency (Table 2). Both conditions (‘ipsi-40 Hz *vs*. ipsi-90 Hz’ and ‘contra-40 Hz *vs*. contra-90 Hz’) revealed insignificant statistical results at the 2000 Hz test frequency. These findings are inconsistent with the results of the previous studies that found lower ASSR thresholds with low MF stimuli.^6^, ^14^ The reason for this dissimilarity is unclear and possibly due to the methodological difference. The present study used narrow band CE-chirp^®^ stimuli for determining ASSR thresholds, whereas pure tones were utilized for recording ASSR thresholds in the previous studies. Since the stimuli used are different, some differences in the study outcomes would be expected.

### Comparison between ipsilateral and contralateral recordings

In the field of ASSR, literatures regarding the influence of ipsilateral and contralateral recordings are limited. In the present study, the majority of test frequencies (500, 1000, and 4000 Hz) found no significant differences in ASSR threshold between ipsi-40 Hz and contra-40 Hz conditions. This suggests that the mode of recording has a subtle influence on ASSR thresholds evoked by low MF stimuli. This finding is in line with the study by Kaf and Danesh^8^ that found no significant differences in ASSR amplitudes and latencies between ipsilateral and contralateral recordings at 500, 2000, and 4000 Hz CFs with 39 Hz MF.

In contrast, for 90 Hz MF stimuli, ipsi-90 Hz condition produced statistically lower ASSR thresholds than that of contra-90 Hz condition at 500, 1000, and 2000 test frequencies. In fact, at these test frequencies, contra-90 Hz condition revealed the highest mean ASSR thresholds. Herein, a significant mode of recording effect on ASSR thresholds is noted for high MF stimuli. This result contradicts high MF findings in the study of Kaf and Danesh.^8^ That is, in their study, the ASSR amplitudes and latencies between ipsilateral and contralateral recordings were not found to be statistically different from each other at all tested CFs with 79 Hz MF. This dissimilarity is possibly due to the methodological difference. While the present study employed ASSR threshold determination, Kaf and Danesh^8^ recorded ASSRs at a supra-threshold level (*i.e.*, 65 dB SPL). As stated earlier, ASSRs recorded at threshold and supra-threshold levels might yield different outcomes.^9^

The superiority of high MF ASSR in recording ipsilateral ASSR thresholds found in the present study is also inconsistent with the study by Small and Stapells.^22^ For adults, they found that the ASSR thresholds recorded at high MFs between ipsilateral and contralateral recordings were not statistically different at 500, 1000, 2000, and 4000 Hz CFs. The reason for this disagreement is perhaps due to difference in sample size. While Small and Stapells^22^ recorded ASSR thresholds in 11 adults, the present study recruited a higher number of participants (*n* = 29). It is known that a larger sample size would increase the statistical power and the likelihood for rejecting the null hypothesis.^23^ In other words, the significant ipsilateral ASSR results obtained for high MF in the present study seem valid due to the larger sample size. Nevertheless, the findings of the current study are consistent with previous studies in infants.^7^, ^22^ Van Maanen and Stapells^7^ determined ASSR thresholds and amplitudes in two age groups of infants (>6 months and ≤6 months) at CFs of 500, 1000, 2000, and 4000 Hz modulated between 81 and 101 Hz. They then found that the contralateral ASSRs showed much smaller amplitudes and were often absent relative to the ipsilateral responses. In line with this, Small and Stapells^22^ found that for infants (mean age of 21 weeks), the ipsilateral ASSR thresholds were significantly lower than the contralateral ASSR thresholds at all tested CFs for both air- and bone-conduction stimulations.

The present study, nonetheless, has some limitations. Firstly, due to the significant interaction effects, the specific effect of test frequency on ASSR thresholds is not determined. Herein, the occurrence of more complex interaction effects was anticipated if frequency is included as one of the factors. Secondly, the present study only tested the right ear of the participants. Since ASSR shows a pronounced laterality effect,^24^ the outcomes of the present study might not be applicable to the left ear. In this regard, future studies are warranted to compare the ASSR thresholds between ears, as well as to further support the relevance of recording mode and modulation frequency in ASSR recording.

## Conclusions

An effort has been made to determine the influences of mode of recording and modulation frequency on ASSR thresholds in young adults. At low frequencies (500 and 1000 Hz), the 40 Hz MF produced lower ASSR thresholds than that of 90 Hz MF. At high frequencies (2000 and 4000 Hz), the ipsi-90 Hz produced the lowest ASSR thresholds. Based on the outcomes of the present study, the authors suggest two different protocols for an optimum threshold determination with ASSR in young adults. In the first protocol, the use of low MF stimuli for recording ASSR thresholds at low frequencies is suggested. At high frequencies, high MF stimuli are recommended. For all conditions, the recording mode is ipsilateral. In the second protocol, the use of low MF stimuli is recommended for determining ASSR thresholds at all test frequencies with contralateral recording.

## Funding

Research University (RU) Grant (1001/PPSK/812114), Universiti Sains Malaysia.

## Conflicts of interest

The authors declare no conflicts of interest.
